# Friend or foe: the role of platelets in acute lung injury

**DOI:** 10.3389/fimmu.2025.1556923

**Published:** 2025-05-14

**Authors:** Jichun Yang, Xun Zhou, Xinrui Qiao, Meng Shi

**Affiliations:** ^1^ Department of Thoracic and Cardiovascular Surgery, Hua Shan Hospital, Affiliated with Fudan University, Shanghai, China; ^2^ School of Integrative Medicine, Tianjin University of Traditional Chinese Medicine, Tianjin, China

**Keywords:** ALI/ARDS, platelets, inflammation, immune regulation, neutrophil, regulatory T cell

## Abstract

Lung diseases, including acute lung injury (ALI) and acute respiratory distress syndrome (ARDS), are associated with various etiological factors and are characterized by high mortality rates. Current treatment strategies primarily focus on lung-protective ventilation and careful fluid management. Despite over 50 years of basic and clinical research, effective treatment options remain limited, and the search for novel strategies continues. Traditionally, platelets have been viewed primarily as contributors to blood coagulation; however, recent research has revealed their significant role in inflammation and immune regulation. While the relationship between platelet count and ALI/ARDS has remained unclear, emerging studies highlight the “dual role” of platelets in these conditions. On one hand, platelets interact with neutrophils to form neutrophil extracellular traps (NETs), promoting immune thrombosis and exacerbating lung inflammation. On the other hand, platelets also play a protective role by modulating inflammation, promoting regulatory T cell (Treg) activity, and assisting in alveolar macrophage reprogramming. This dual functionality of platelets has important implications for the pathogenesis and resolution of ALI/ARDS. This review examines the multifaceted roles of platelets in ALI/ARDS, focusing on their immunomodulatory effects, the platelet-neutrophil interaction, and the critical involvement of platelet-Treg cell complexes in shaping the inflammatory environment in ALI.

## 1 Introduction

Acute lung injury (ALI) and acute respiratory distress syndrome (ARDS) present clinically as rapid-onset respiratory failure, with a mortality rate reaching approximately 40% in severe cases (1, 2). Recent epidemiological studies estimate the global incidence of ARDS to range from 10 to 86 cases per 100,000 people per year, depending on population demographics and diagnostic criteria (3, 4). Despite advances in intensive care, ARDS remains associated with substantial morbidity and mortality, with hospital mortality rates ranging from 30% to over 40%, particularly in moderate-to-severe cases (5). Even with optimal supportive care, a major clinical trial reported a 42.8% mortality rate by day 90 in patients with moderate-to-severe ARDS, underscoring the urgent need for improved therapeutic strategies (6). A variety of precipitating factors, including severe infections, inhalational lung injuries, ischemia-reperfusion events, multi-trauma, extensive blood transfusions, and acute pancreatitis, can directly or indirectly damage lung tissue or associated vasculature, often serving as precursors to the rapid onset of respiratory failure (7). A marked increase in ARDS cases was observed during the coronavirus disease 2019 (COVID-19) pandemic, caused by severe acute respiratory syndrome coronavirus 2 (SARS-CoV-2), underscoring ARDS as a critical global health challenge (8).

The concept of ALI and ARDS was first introduced by Ashbaugh et al. in 1967 (9). In 1994, a collaboration between European and American experts established diagnostic criteria for ALI and ARDS, specifying the presence of acute bilateral lung infiltrates on chest imaging, no evidence of elevated left atrial pressure, and a ratio of arterial oxygen partial pressure to fractional inspired oxygen (PaO2/FiO2) ≤ 300 mmHg for ALI or < 200 mmHg for ARDS (10, 11). The Berlin definition, introduced in 2012, further refined these parameters by standardizing the grading of ARDS severity based on oxygenation levels: mild (200 < PaO2/FiO2 ≤ 300 mmHg), moderate (100 < PaO2/FiO2 ≤ 200 mmHg), and severe (PaO2/FiO2 ≤ 100 mmHg). This updated framework also provided objective diagnostic tools and emphasized clinical markers, addressing limitations in the earlier US-European Consensus Conference criteria (12). Despite these advancements, the clinical mortality rate for ALI and ARDS remains alarmingly high, with treatment options largely limited to supportive care. Current approaches focus primarily on protective mechanical ventilation, corticosteroid-assisted fluid management, and interventions targeting the underlying cause (13, 14). However, even with optimal supportive care, a major clinical trial reported a 42.8% mortality rate by day 90 in patients with moderate-to-severe ARDS (15). Although mechanical ventilation is indispensable, it is associated with risks such as ventilator-induced ALI (VILI), which exacerbates inflammation and may increase the likelihood of pulmonary fibrosis (16, 17). These challenges highlight the need for a deeper understanding of the complex pathogenesis of ALI and ARDS, as well as for developing novel therapeutic strategies.

Recent studies suggest that ARDS is not a single disease entity but rather a syndrome composed of distinct phenotypic subgroups that exhibit different inflammatory responses, clinical outcomes, and treatment responses (18, 19). While traditional clinical trials have treated ARDS as a homogeneous condition, emerging evidence supports the need for a phenotype-driven approach to improve patient selection and therapeutic efficacy. Genome-wide association studies (GWAS) have identified platelet count as a key intermediate phenotype in ARDS, linking platelet activation to disease severity and genetic susceptibility (20). Moreover, phenotypic variability has significantly influenced the success rates of clinical trials evaluating novel ARDS treatments, highlighting the necessity of patient enrichment strategies to enhance study design and improve therapeutic targeting (21). Integrating genetic and phenotypic stratification into ARDS research and clinical management may lead to more precise and effective interventions, ultimately improving patient outcomes.

Excessive inflammatory responses are central to the pathogenesis of ALI and ARDS, leading to a massive influx of neutrophils, macrophages, and other inflammatory cells that cause extensive damage to pulmonary vascular endothelial and alveolar epithelial barriers (22, 23). Platelets, long recognized for their role in coagulation, have recently emerged as key mediators of inflammation and immune regulation (24). Increasing evidence suggests that platelets interact closely with neutrophils to form platelet-neutrophil complexes, which amplify inflammatory cascades and contribute to tissue damage (25). For instance, Semaphorin 7A (Sema7A), a glycosylphosphatidylinositol-anchored protein, interacts with PlexinC1 receptors on neutrophils, enhancing neutrophil activation, chemotaxis, and adhesion (26). This interaction facilitates the formation of platelet-neutrophil aggregates, which promote the release of neutrophil extracellular traps (NETs), a process closely linked to endothelial cell injury and alveolar-capillary barrier disruption (26, 27). NETs, though initially protective by capturing pathogens, release cytotoxic histones and proteases that exacerbate pulmonary inflammation, edema, and tissue damage (28). The Sema7A-PlexinC1 axis has also been shown to enhance neutrophil transmigration across the endothelial barrier, further reinforcing the role of platelet-neutrophil interactions in ALI and ARDS (26).

Platelets store and release a wide range of inflammatory mediators, including cytokines and chemokines, which propagate the immune response upon activation (29). For example, activated platelets release factors such as P-selectin, platelet factor 4 (CXCL4), and interleukin-1β (IL-1β), which recruit neutrophils to sites of injury and amplify leukocyte aggregation (29). Platelet-endothelial interactions also exacerbate inflammation by increasing vascular permeability and disrupting the alveolar-capillary barrier, further contributing to pulmonary edema and impaired gas exchange (30). Consequently, platelets are often regarded as amplifiers of ALI and ARDS, driving hyperreactive inflammatory cascades (31). However, in addition to their well-characterized pro-inflammatory role, platelets are increasingly recognized as key regulators of immune balance through their interactions with various immune cells, including regulatory T cells (Tregs) and alveolar macrophages (30, 32). These interactions contribute not only to modulating inflammatory responses but also to promoting tissue repair and resolution of ALI. While the immunoregulatory functions of platelets have gained attention in recent years, the precise molecular mechanisms governing these processes remain incompletely understood, highlighting the need for further investigation. Earlier reviews, such as the work by Middleton et al. (2018) (33), have provided a detailed discussion of platelet involvement in ALI/ARDS, particularly emphasizing their role in inflammation and thrombosis. Our review extends this discussion by incorporating recent findings that shed light on platelet-driven immune modulation. Specifically, we examine how platelet interactions with Tregs and macrophages shape the inflammatory milieu and influence lung recovery. Furthermore, our work takes a more translational perspective by exploring a broader range of antiplatelet and immunomodulatory therapies, assessing their potential use across different ARDS phenotypes. In addition, we discuss the emerging significance of lung-resident megakaryocytes and their potential contributions beyond platelet production, offering new insights into their role in both local and systemic immune regulation within the context of ALI/ARDS. Emerging evidence suggests that targeting platelets may provide therapeutic benefits. For instance, antiplatelet therapies such as aspirin have demonstrated the ability to attenuate hyperoxia-induced ALI by modulating platelet-driven inflammatory pathways (34, 35). Additionally, platelet glycoprotein VI (GPVI) has been identified as a key mediator of neutrophil recruitment, migration, and NETosis in early ALI and ARDS (36). Novel therapeutic approaches, such as tea polyphenol-loaded nanoparticles coated with platelet membranes, have shown promise in ameliorating lipopolysaccharide (LPS)-induced ALI, further highlighting platelets as a potential therapeutic target (37).

Interestingly, platelets exhibit a dual role in ALI and ARDS (38). In the early stages, platelets amplify inflammation by releasing pro-inflammatory mediators, while in the later stages, they contribute to the resolution of inflammation (39). In infectious pneumonia, platelets maintain the alveolar-capillary barrier and reduce the virulence of pathogens, thereby protecting against severe pulmonary complications (40, 41). During the resolution phase, platelets promote macrophage polarization from the pro-inflammatory M1 phenotype to the anti-inflammatory M2 phenotype, facilitating tissue repair and recovery (42, 43). Furthermore, platelets interact with regulatory T cells (Tregs), enhancing their activation and secretion of anti-inflammatory cytokines such as transforming growth factor-beta (TGF-β) and interleukin-10 (IL-10) (44). These interactions are crucial for suppressing excessive inflammation and promoting the clearance of apoptotic neutrophils (41, 42). This dual functionality raises critical questions: What molecular signals govern the transition of platelets from pro-inflammatory to anti-inflammatory states? How do platelets interact with other immune cells to balance inflammation and tissue repair? Addressing these questions may provide insights into novel therapeutic strategies for ALI and ARDS.

## 2 Activated platelets are essential inflammatory and immune effector cells in ALI/ARDS

Platelets, anucleate cells derived from megakaryocytes, are second only to red blood cells in abundance within circulation. Historically, platelets were thought to originate exclusively from bone marrow megakaryocytes. Recent studies have revealed that lung-resident megakaryocytes also actively produce circulating platelets, adding an important dimension to their biology (45, 46). Platelets are crucial for physiological hemostasis and pathological thrombosis, as they rapidly adhere to damaged vessel walls, form aggregates, and initiate clot formation (47, 48). Damage to the vascular wall triggers platelets to release procoagulant factors, recruiting leukocytes and red blood cells to form a thrombotic barrier at the injury site, preventing further bleeding and microbial invasion (32, 49).

Beyond hemostasis, platelets are now recognized as key players in immune responses. They interact with various immune cells, such as neutrophils and macrophages, releasing bioactive molecules from intracellular granules (50, 51). These molecules act to bridge innate and adaptive immunity, positioning platelets as critical drivers of inflammation, particularly in the pathogenesis of ALI/ARDS (51–53). Platelets fine-tune immune responses by modulating the phenotype and activity of immune cells, thereby influencing the progression and resolution of ALI (54).

### 2.1 Platelet surface adhesion molecules and receptors

Platelet activation is a multistep process triggered by vascular injury or inflammation. Once activated, platelets adhere to neutrophils, endothelial cells, and other immune cells, forming platelet-leukocyte aggregates that amplify the inflammatory response (55, 56). Platelets recognize von Willebrand factor (VWF) and collagen at the site of injury through specific receptors, such as glycoproteins (GP) Ib-IX-V, GPVI, and GPIIb/IIIa (57–59). These interactions not only promote adhesion to the endothelium but also initiate rapid intracellular signaling that stabilizes thrombus formation and amplifies inflammation (60, 61).

A key mediator of platelet-neutrophil interactions is P-selectin, which binds to P-selectin glycoprotein ligand-1 (PSGL-1) on neutrophils. This interaction triggers signaling cascades, such as extracellular signal-regulated kinase (ERK)1/2- mitogen-activated protein kinase (MAPK), activating neutrophil integrins like Mac-1 and LFA-1 (30, 62). These integrins facilitate neutrophil adhesion and migration across the endothelium, further amplifying the inflammatory response in ALI/ARDS (63, 64). The formation of platelet-neutrophil aggregates (PNAs) enhances neutrophil activation, leading to ROS production and NETs release, which exacerbate endothelial dysfunction and alveolar injury (65, 66), as described earlier.

Recent studies have highlighted the role of mitochondrial dynamics in platelets, showing that mitofusin-2 (Mfn2) regulates platelet-neutrophil interactions by influencing mitochondrial ROS production (67, 68). Dysregulation of this pathway exacerbates platelet-neutrophil aggregate formation, worsening inflammation and ALI (67). Moreover, platelet GPIIb/IIIa binds to soluble fibrinogen, creating a bridge with neutrophil Mac-1, further stabilizing platelet-neutrophil complexes during inflammation (64, 69). Additionally, P-selectin stored in platelet alpha granules and endothelial Weibel-Palade bodies mediates platelet-leukocyte adhesion, making it a key player in ALI (70).

Therapeutic strategies targeting these interactions, such as P-selectin inhibitors and GPVI signaling blockers, have shown promise in preclinical models (71, 72). These approaches reduce platelet-neutrophil aggregates, mitigate NET formation, and alleviate inflammation, offering potential avenues for ARDS treatment (63).

### 2.2 Platelets are rich in a variety of immune mediators

Platelets are small anucleate cells that store and release various thrombosis and immune regulation-associated bioactive substances (**Table 1**). These molecules, packaged in alpha granules, dense granules, and lysosomal granules, are released upon platelet activation in response to vascular injury or inflammatory stimuli (83).

**Table 1:** Important platelet-derived immune mediators.

Category	ingredient		Functions
Alpha granules	Integrins	αIIbβ3	Initiates bidirectional signal transduction promotes platelet activation (55)
	Chemokines	PF4 (CXCL4)	Aggravates pulmonary fibrosis by stimulating endothelial cells to transform into mesenchymal tissue
		RANTES (CCL5)	T and B cell responses and development (27)
	Adhesion molecule	GPIB/IX/V complex	Binds to P-selectin and mediates platelet adhesion and aggregation; binds to vWF and activates αIIbβ3 (73)
		P-selectin (CD62P)	Its binding to PSGL-1 on neutrophils initiates the platelet-neutrophil interaction (74)
		PECAM-1 (CD31)	As an adhesive stress response protein, it maintains endothelial cell integrity and accelerates the recovery of vascular barriers
		TLT-1 receptor	Enhances platelet aggregation and mediates interactions with neutrophils and endothelial cells
	Cytokines	IL-1I (IL-1α, L-1β)	Platelets affect the IL-1 production in the body and regulate it to drive inflammation
	Growth factors	TGF-β	It regulates fibroblast recruitment to the site of lung tissue injury and promotes lung tissue repair (75)
		PDGF	Promote damage repair and differentiation and proliferation of VSMC
	TLR	Surface TLRS	TLR2and TLR4	Recognizes surface protein components of pathogens, amplifies platelet activation (76) and aggregation, and induces NETs formation (77)

			Endosomal TLRs	TLR7	Receptors for single-stranded viral RNA
	TLR9	Platelet TLR9 is a functional platelet receptor that links oxidative stress, innate immunity, and thrombosis (78)		
δ-granule molecules	Amines and mediators	Thrombin	In patients with COVID-19, platelet thrombin is associated with alveolar-capillary microthrombosis (79)
		5-HIAA	Acts on the GPR35 ligand of neutrophils to promote neutrophil migration to damaged tissue (80)
	Nucleotides	ADP	Recruitment of activated platelets and exposure of P-selective velocity (81)
Lysosomes	Glycohydrolases	Heparinase	A lytic enzyme that can induce thrombocytopenia
Surface protein expression	CLEC-2		Termination of signaling by PMN recruited in the early stages of acute lung inflammation (82)

5-HIAA, 5-hydroxyindole acetic acid; ADP, adenosine diphosphate; CCL5, Chemokine (C-C motif) ligand 5; CLEC-2, C-type lectin-like receptor; GPIB, glycoprotein IB complex; gpr35, G protein-coupled receptor; HAG, hetero-aggregate; IL-1, interleukin 1; NETs, neutrophil extracellular traps; PDGF, platelet-derived growth factor; PDPN, podoplanin; PECAM-1, Platelet/endothelial cell adhesion molecule-1; PF4, Platelet factor 4; PMN, polymorphonuclear leukocytes; PRRS, pattern recognition receptors; PSGL-1, P-selectin glycoprotein ligand-1; TGF-β, transforming growth factor-beta; TLR1-9, toll-like receptor 1-9; TLT-1, Trem-like transcript 1; vWF, von Willebrand factor; VSMC, vascular smooth muscle cell.

Alpha granules are particularly important for inflammation and immune regulation, as they contain mediators such as platelet factor 4 (PF4), RANTES, and interleukin-8 (IL-8). These mediators play key roles in recruiting neutrophils and other leukocytes to sites of injury (84–86). PF4, a member of the CXC chemokine family, exhibits dual roles in promoting neutrophil recruitment and modulating immune responses. Elevated PF4 levels have been correlated with disease severity in ARDS, underscoring its clinical relevance (84, 87).

Dense granules store small molecules, including ADP, ATP, and serotonin (5-HT), which amplify platelet activation through feedback mechanisms involving the P2Y12 receptor (88). Recent research has identified 5-hydroxyindoleacetic acid (5-HIAA), a serotonin metabolite released by activated platelets, as a ligand for G protein-coupled receptor 35 (GPR35) (89, 90). This signaling axis promotes neutrophil migration and adhesion, highlighting the intricate role of platelet-derived mediators in inflammation (80). Lysosomes, though less studied, contain acid hydrolases and glycohydrolases that dissolve platelet aggregates, helping regulate thrombosis and inflammation (91).

Although platelets lack nuclei, they retain functional mRNA and splicing machinery, allowing them to synthesize proteins such as interleukin-1 beta (IL-1β) in response to stimuli (92, 93). This dynamic capability enables platelets to adapt to changing inflammatory environments. Alterations in platelet transcriptomes, observed in COVID-19 patients, have revealed upregulation of inflammatory pathways, such as MAPK signaling, linked to severe ALI (94).

Platelet-derived extracellular vesicles (EVs), including exosomes and microvesicles, are another important mechanism by which platelets influence inflammation (95). These vesicles carry cytokines, chemokines, and nucleic acids, modulating endothelial barrier function and reducing inflammation in preclinical ALI models. Recent advancements in biomimetic nanoparticle technology have demonstrated the potential of platelet-derived vesicles as targeted drug delivery systems, offering new therapeutic opportunities (94, 96–100).

## 3 Platelets promote neutrophil recruitment, pathogen elimination, and induction of neutrophil extracellular traps release in ALI/ARDS

**Figure 1:**
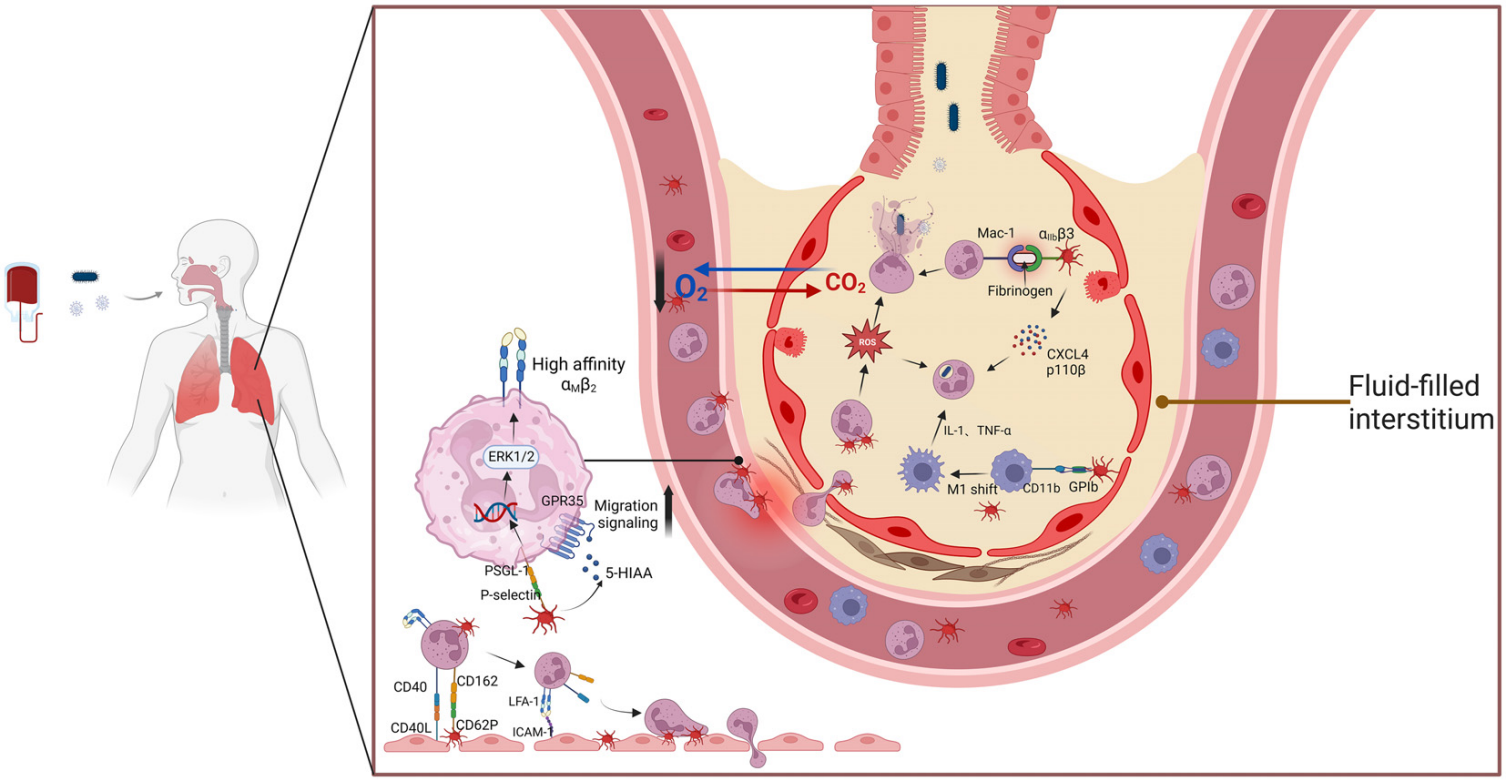
The interaction between platelets and neutrophils aggravates ALI/ARDs. This schematic illustration highlights the critical role of platelet-neutrophil interactions in the pathogenesis of ALI and ARDS. Upon activation, platelets release inflammatory mediators such as PF4, P-selectin, and CCL5, which facilitate neutrophil recruitment and adhesion. The formation of platelet-neutrophil aggregates enhances NET release, a process that contributes to endothelial damage, increased vascular permeability, and alveolar inflammation. Additionally, interactions between PSGL-1 on neutrophils and P-selectin on platelets promote immune cell crosstalk, amplifying inflammatory responses. Excessive NET formation releases cytotoxic histones and proteases that disrupt the alveolar-capillary barrier, exacerbating lung injury. These mechanisms collectively contribute to the progression of ARDS, underscoring the potential of targeting platelet-neutrophil interactions as a therapeutic strategy. Abbreviations: ALI, acute lung injury; ARDS, acute respiratory distress syndrome; PF4, platelet factor 4; NETs, neutrophil extracellular traps; PSGL-1, P-selectin glycoprotein ligand-1; CCL5, chemokine (C-C motif) ligand 5.

The activation and recruitment of neutrophils are essential processes in the pathogenesis of ALI (ALI) and acute respiratory distress syndrome (ARDS) (101). These processes are closely associated with platelet-neutrophil interactions during the inflammatory surge in ALI/ARDS. Platelet activation enhances neutrophil recruitment through P-selectin-PSGL-1 interactions and promotes the formation of platelet-neutrophil aggregates (PNAs). This interaction amplifies neutrophil activation and inflammatory mediator release, contributing to endothelial damage and alveolar injury (**Figure 1**) (102, 103). Evidence from lipopolysaccharide (LPS)-induced pneumonia models suggests that platelet depletion leads to a significant reduction in neutrophil recruitment, highlighting the platelet’s indispensable role in coordinating the inflammatory response (104). Furthermore, studies have demonstrated that inhibiting platelet-derived chemokines such as CCL5 and CXCL4 effectively prevents the progression of ALI (105). In addition, platelet integrin-mediated signaling inhibition reduces NET release, thereby mitigating lung tissue damage in ALI VILI models (106).

Platelet P-selectin stored in alpha granules plays a key role in mediating platelet-neutrophil interactions. Blocking P-selectin has been shown to diminish platelet-neutrophil aggregates and slow ALI progression in acid-induced ALI models (65). Protease-activated receptor 2 blockade has also been reported to inhibit carbamoyl-platelet-activating factor (PAF)-mediated neutrophil recruitment and inflammation in mouse lung tissue (107). Furthermore, cigarette smoke-induced severe influenza has been shown to worsen due to platelet-driven pulmonary microvascular occlusion, underscoring the role of platelet-neutrophil aggregation in exacerbating ALI (108).

### 3.1 Platelets and neutrophil recruitment in ALI/ARDS

Physiologically, red and white blood cells are primarily located in the central vascular region, while platelets are concentrated near the vascular endothelium, positioning them to interact with leukocytes under both physiological and pathological conditions (109). Even in the absence of inflammation, transient interactions occur between platelets and neutrophils near the vascular endothelium (110, 111). Once activated, platelets regulate neutrophil rolling and adhesion, which are critical for neutrophil recruitment during inflammation, as demonstrated in various pneumonia models (65).

In inflammatory environments, platelets act as navigators, bridging neutrophils and endothelial cells. P-selectin on platelets interacts with neutrophil P-selectin glycoprotein ligand-1 (PSGL-1), mediating high-affinity activation of neutrophil integrin β2 via ERK1/2 MAPK signaling. This activation enhances neutrophil adhesion and transmigration across endothelial barrier (112). Integrins such as LFA-1 and Mac-1 further stabilize platelet-neutrophil complexes and promote neutrophil extravasation (113, 114).

Besides direct interactions, platelets also maintain vascular endothelial integrity indirectly through mechanisms involving phosphoinositide 3-kinase (PI3K) isoforms, particularly p110β. In pneumococcal pneumonia-induced mouse ALI models, p110β promotes platelet activation, neutrophil extravasation, and bacterial clearance (115). These findings underscore the indispensable role of platelets in coordinating neutrophil recruitment and regulating vascular inflammation.

### 3.2 Neutrophil functions: reactive oxygen species and phagocytosis

Neutrophils play critical roles in pathogen elimination and the regulation of pulmonary injury (116, 117). ROS generated by neutrophils are essential for microbial killing but can exacerbate tissue injury when excessively produced (118). Platelet-neutrophil aggregates amplify ROS production. Specifically, studies have shown that when neutrophils bind to platelets on immunoglobulin G (IgG)-coated surfaces, ROS release is significantly increased (119). ROS also influences specific pathways involved in NET formation, further enhancing pathogen clearance capacity (120). Platelets further enhance neutrophil phagocytosis through signaling pathways such as TLR2/PI3K/AKT (121). These pathways facilitate bacterial engulfment by neutrophils, though the detailed molecular mechanisms remain a promising area for future investigation (122).

### 3.3 NET formation and its dual role in ALI/ARDS pathogenesis

**Figure 2:**
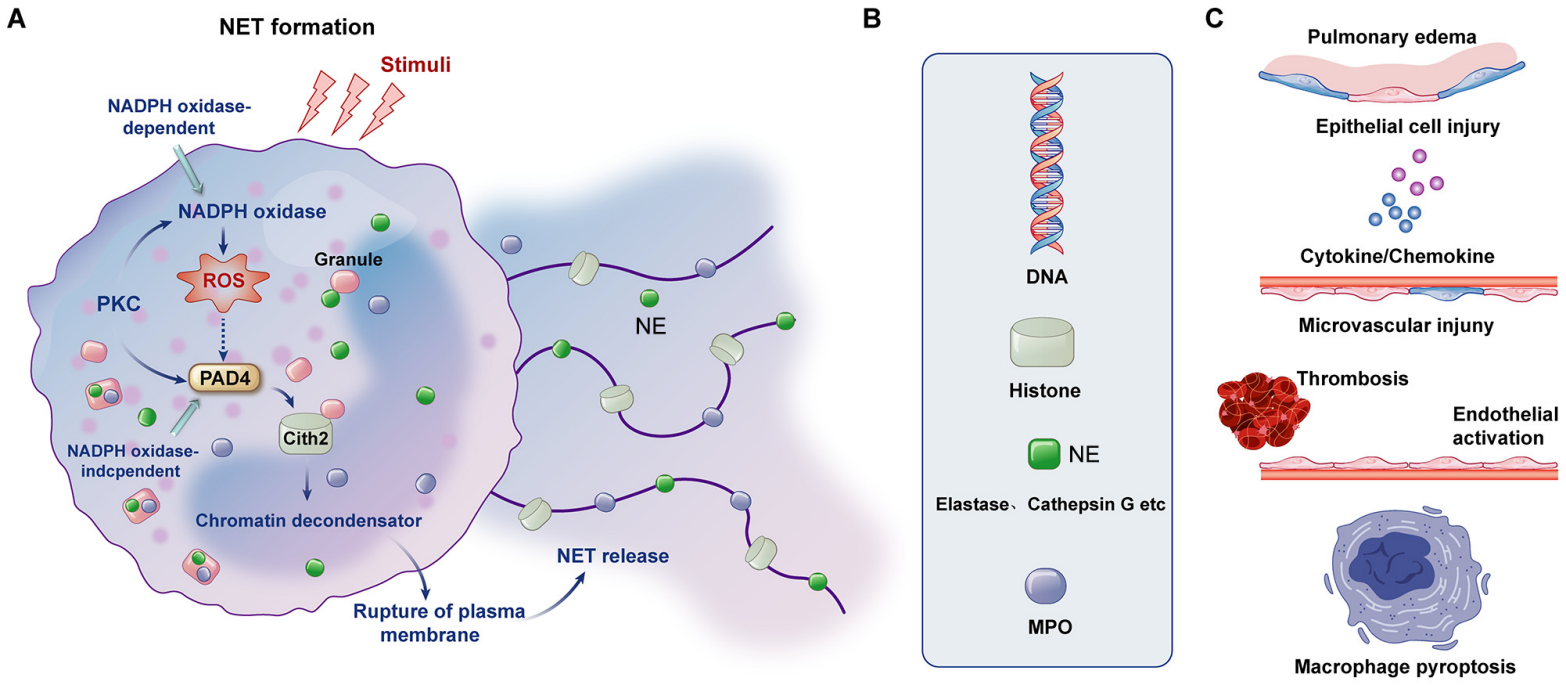
Formation of NETs, components of NETs and mechanisms affecting ARDS. **(A)** The formation of NETs can be divided into NADPH oxidase-dependent and independent formations. **(B)** The major components of NETs include DNA, histones, NE and MPO. **(C)** NETs and their components can cause pulmonary tissue edema, damage alveolar epithelial cells and microvessels, cause the formation of immune thrombosis and activation of endothelial cells, and mediate the pyroptosis of macrophages and the release of downstream cytokines.

NETs, composed of DNA scaffolds modified by histones, myeloperoxidase (MPO), and neutrophil elastase (NE), serve as robust antimicrobial barriers (123). Dysregulated NET formation contributes to aseptic inflammation, thrombosis inflammation, and tissue injury in ALI/ARDS (**Figure 2**) (124, 125). NET production is primarily driven by ROS generated through NADPH oxidase, which stimulates MPO and NE activity, leading to chromatin decondensation (126). Pore-forming proteins like gasdermin D facilitate the extracellular release of NETs in the NADPH oxidase 2 (NOX2)-ROS-dependent pathway (127).

During pulmonary infection, platelet-neutrophil complexes critically modulate NET release through multiple mechanisms. These include PSGL-1 signaling, platelet glycoprotein VI (GPVI) signaling, and high mobility group box 1 (HMGB1)-mediated autophagy (64, 128). Platelet P-selectin also promotes NET release by binding to PSGL-1, while fibrinogen-mediated interactions between platelet integrin β3 and neutrophil Mac-1 further enhance NET formation (129). Inhibition of GPVI signaling has shown promise in experimental LPS-induced ALI models by reducing NET release and mitigating lung inflammation (37).

Therapeutic strategies targeting platelet-mediated NET formation represent a potential avenue for ALI/ARDS treatment. For example, GPVI signaling inhibitors and HMGB1-targeting therapies have demonstrated efficacy in reducing NET-mediated tissue damage in experimental models (130).

### 3.4 Concluding remarks on platelet-neutrophil interactions in ALI/ARDS

Severe infection, inhalation injury, and massive blood transfusion are significant risk factors for ALI and ARDS. In these contexts, platelets play pivotal roles in neutrophil recruitment and NET formation, processes that are central to the inflammatory and immune responses in ALI/ARDS. For instance, platelet P-selectin-PSGL-1 interactions promote neutrophil adhesion and migration to injured alveoli. Platelet-derived chemokines, including CXCL4 and p110β, enhance neutrophil phagocytic capacity, aiding in bacterial clearance (106). Moreover, platelet-neutrophil complexes amplify ROS production and NET release, further influencing the disease’s progression.

While NETs serve protective roles by limiting pathogen spread, their dysregulated release can exacerbate tissue damage and promote immunothrombosis. Therefore, therapeutic strategies that modulate platelet-neutrophil interactions, particularly NET formation, hold promise for mitigating inflammation and improving clinical outcomes in ALI/ARDS. Future research should aim to elucidate the precise molecular mechanisms underlying these interactions and identify novel therapeutic targets.

## 4 Activated platelets, endothelial cells, and neutrophils mixed results in the formation of immunothrombosis aggravate ALI/ARDS

Innate immunity serves as the first line of defense against pathogens. Platelets play a crucial role not only in directly eliminating microorganisms but also in mediating the formation of immune thrombosis, a process referred to as “immunothrombosis” (131). Unlike classical thrombosis, activated platelets can interact with neutrophils and complement proteins to trigger the coagulation cascade, forming thrombi within microvessels. This disruption of the balance between coagulation and inflammation leads to severe inflammatory responses, resulting in widespread damage to pulmonary capillaries and alveolar edema (132). The complex formation of immunothrombosis involves the activation of platelets, endothelial cells, neutrophils, NETs, and microparticles, all of which are recognized as key contributors to the pathogenesis of ALI/ARDS [119]. In the case of COVID-19 caused by SARS-CoV-2, nearly 25% of patients with severe disease exhibit pronounced hypercoagulability (133). Postmortem lung tissue analyses from COVID-19 patients have consistently revealed the presence of disseminated microthrombi (134–136).

### 4.1 Role of endothelial cells in ALI/ARDS-related inflammation

ALI is characterized by intricate interactions among immune cells, inflammatory mediators, and tissue components (137). The progression from acute inflammation to healing involves a series of coordinated cellular pathways (138). Initially, during ALI, alveolar epithelial cells are activated by pro-inflammatory cytokines, which leads to an increase in vascular permeability and allows immune cells, especially neutrophils, to infiltrate the lung tissue (139). This inflammatory cascade is regulated by signaling pathways such as NF-κB and MAPK, with key inflammatory mediators like TNF-α and IL-1β playing crucial roles (140, 141).

An intact endothelial barrier is crucial for maintaining vascular permeability and ensuring the diffusion of nutrients, oxygen, and metabolic waste products (142). In the context of ALI/ARDS, endothelial cells regulate vascular permeability, and platelet activation and aggregation can lead to the formation of microthrombi (38). Endothelial cells are covered by a glycocalyx, a multi-layered structure that limits direct contact between endothelial cells and blood components, thereby inhibiting leukocyte and platelet adhesion, coagulation, and microthrombosis (136, 143). However, this protective barrier is compromised in the inflammatory environment of ARDS (144).

ALI/ARDS is a life-threatening lung condition characterized by the disruption of the alveolar-capillary barrier, leading to pulmonary edema and impaired gas exchange (145). Endothelial cell injury and inflammation are central to the development of ALI/ARDS (146). As a highly dynamic and metabolically active tissue, the endothelium plays a critical role in maintaining organ homeostasis (147). During inflammation, endothelial cells are among the first to respond to inflammatory stimuli (148). In ALI/ARDS, the activation of inflammatory cells (e.g., neutrophils) and the release of inflammatory mediators (e.g., TNF-α, interleukins) exacerbate endothelial cell injury (149). This endothelial damage results in the increased release of inflammatory mediators, which further attract additional immune cells, creating a vicious cycle of sustained inflammation. Consequently, endothelial injury increases the permeability of the blood vessel wall, promoting the development of pulmonary edema and impairing gas exchange, ultimately leading to symptoms such as dyspnea and hypoxemia (150). Recent studies suggest that lung endothelial cells are key regulators of both innate and adaptive immunity, playing an essential role in the pathogenesis of ARDS (142). Additionally, endothelial cell vesicles (ECVs) have been shown to exacerbate ALI/ARDS by transmitting inflammatory signals. These vesicles carry FSTL1, which activates inflammatory pathways like the TLR4/JAK3/STAT3/IRF-1 pathway (151). Moreover, TRIM47 has been implicated in enhancing the inflammatory response and promoting endothelial activation in ALI/ARDS (152).

### 4.2 Interaction between activated platelets and endothelial cells

Activated platelets interact with endothelial cells to induce immune responses that promote immunothrombosis. Specifically, IL-1β released by activated platelets stimulates endothelial cells, increasing their permeability and accelerating the extravasation of fluid and proteins (153). Additionally, activated platelets bind to endothelial cell PSGL1 via P-selectin, while glycoprotein GPIb interacts with von Willebrand factor on endothelial cells (154). This interaction facilitates the migration of inflammatory cells, thereby promoting the formation of immunothrombosis (155, 156). Clinical manifestations of ARDS, such as endothelial dysfunction and immune-thrombosis-related complications, are attributed to this complex interplay between platelets and endothelial cells (142, 157, 158). Flow chamber analyses have shown that platelet-endothelial interactions are critical for maintaining vascular integrity and regulating blood flow (159).

### 4.3 Activated platelets mediate the formation of NETs, promoting immunothrombosis

Neutrophils are central to the early stages of ALI, as they release ROS and proteolytic enzymes that damage both the endothelial and epithelial barriers (101). However, the unchecked activation of neutrophils can worsen the condition by sustaining the inflammatory response and causing further tissue damage (138). Beyond their traditional role in clot formation, platelets also contribute significantly to the regulation of inflammation (160). The interaction between platelets and neutrophils, mediated by P-selectin, amplifies the inflammatory response by enhancing neutrophil recruitment and promoting the formation of NETs (161).

As ALI progresses, activated platelets express P-selectin, which binds to neutrophil PSGL-1 to promote the formation of NETs, structures that trap pathogens (162). However, in this process, the presence of tissue factor within NETs stimulates the release of thrombin, which in turn activates platelets, initiating a vicious cycle of immune thrombosis and potentially leading to disseminated intravascular coagulation (DIC) (163, 164). Activated platelets also secrete various proinflammatory molecules, including soluble P-selectin, platelet factor 4 (PF4), platelet-activating factor, and neutrophil-activating peptides, which promote neutrophil recruitment to sites of thrombosis (131, 165). Furthermore, high mobility group box 1 (HMGB1) released from activated platelets contributes to NET formation. HMGB1 interacts with the receptor for advanced glycation end products (RAGE) on neutrophils, promoting autophagy and NET generation, independent of NADPH oxidase-mediated ROS production (130, 166, 167). Platelet TLR4 signaling plays a pivotal role in this process. When LPS binds to TLR4, activated platelets interact with neutrophils through GP1b and neutrophil integrin β2, facilitating NET formation (66, 168). In experimental models of transfusion-associated ALI, the inhibition of platelet activation via aspirin or GPIIb/IIIa blockers has been shown to reduce NET formation and alleviate ALI (169, 170). The release of tissue factor (TF) from NETs further amplifies the imbalance between inflammation and coagulation, exacerbating immunothrombosis and leading to poor ALI prognosis (164). This process is heavily influenced by the activation of the STING (stimulator of interferon genes) pathway and TLR2 on endothelial cells (171). In summary, activated platelets not only induce NET formation but also recruit platelets to vascular sites under high shear conditions, triggering further platelet activation and perpetuating a cycle of immunothrombosis (172).

## 5 Investigation of the role of platelets in regulating the resolution of inflammation in patients with ALI/ARDS

Damage to tissues or microbial invasion triggers an acute response to protect the host. However, excessive and prolonged acute inflammation can cause tissue damage and impair organ function, ultimately leading to disease. To limit inflammation and prevent collateral damage to healthy tissue, lung tissue orchestrates the formation of specific pro-resolving mediators, such as lipoxins, protectins, and maresins (173). These mediators act at specific nodes to prevent further leukocyte recruitment, promote apoptosis of neutrophils, eliminate apoptotic cells, convert macrophages from a pro-inflammatory to a pro-resolving phenotype, and inhibit pro-inflammatory mediators, ultimately restoring homeostasis (174, 175). Platelets play a crucial role in controlling the resolution of inflammation in ALI/ARDS by influencing macrophage activity, T cells, and the secretion of anti-inflammatory mediators (176, 177). Consequently, research on platelet immune function is increasingly focusing on their role in pro-resolution rather than in promoting inflammation (178).

### 5.1 Role of platelets in macrophage polarization

Macrophages and T regulatory (Treg) cells are essential in the resolution of inflammation in ALI (179). To restore balance, the effector function of macrophages shifts from a pro-inflammatory (M1) to an anti-inflammatory (M2) phenotype (180–182). During ALI, M1 macrophages initially take the lead in guiding neutrophils to eliminate pathogens (183). Platelets, in association with GPIb-CD11b interactions, promote monocyte-mediated M1 macrophage polarization (184). As ALI progresses into the resolution phase, M2 macrophages dominate, regulating the proliferation and differentiation of alveolar type 2 (AEC2) cells, thus promoting lung tissue repair (185, 186). These M2 macrophages secrete prostanoids like PGE2, which regulate the production of anti-inflammatory lipoxin A4, downregulate CXCR2 expression, reduce ROS levels, inhibit polymorphonuclear neutrophil (PMN) migration, and prevent the release of NETs (187).

The two types of pulmonary alveolar macrophages (AMs) are monocyte-derived alveolar macrophages (Mo-AMs) and tissue-resident alveolar macrophages (TR-AMs) (188). Compared to TR-AMs, Mo-AMs are more plastic and derived from monocytes that enter the alveoli after ALI (189, 190). The properties and functions of Mo-AMs depend on the regulation of the lung microenvironment. Early inflammatory Mo-AMs exhibit the M1 phenotype, exerting significant pro-inflammatory effects that can exacerbate tissue injury (191). During the resolution phase, Mo-AMs adopt a transcriptional profile favoring tissue repair (192–194). TR-AMs, which are the “guardians” of the alveoli, effectively recognize and absorb inhaled pathogens (195). TR-AMs also support the termination and resolution of ALI inflammation through mechanisms potentially driven by β-catenin-hypoxia inducible factor-1α signaling (196). TR-AMs also release anti-inflammatory mediators like transforming growth factor beta (TGF-β) and IL-10, facilitating tissue repair (197, 198).

Recent studies have shown that ALI can trigger antigen-specific CD4+ T cell activation, amplifying Treg regulatory function during acute tissue injury (199). CD4+ T cells differentiate based on the signals they receive (200). For example, IL-2 and Janus kinase (JAK) tyrosine kinases initiate T-cell receptor (TCR) signaling, engaging signal transducer and activator of transcription 5 (STAT5) and Foxp3, leading to Treg differentiation (201, 202). Tregs regulate neutrophil apoptosis, prevent neutrophil migration, and promote lung tissue repair by releasing TGF-β (203, 204). Additionally, Tregs stimulate the proliferation and differentiation of AEC2 and directly aid in the regeneration of damaged alveolar epithelial cells (205–207). In contrast, IL-6 promotes Th17 differentiation through the JAK/STAT3/RORγt pathway, exacerbating ALI by releasing the pro-inflammatory cytokine IL-17A (208–210). The synergistic interaction between macrophages and Tregs promotes ALI resolution. Macrophages regulate the Th17/Treg balance in ALI, increasing the number of anti-inflammatory cells during the resolution phase, while Tregs guide macrophages toward the M2 phenotype (179, 211). Tregs also enhance macrophage phagocytic capacity by secreting IL-13 and stimulating macrophages to release anti-inflammatory cytokines such as TGF-β and IL-10 (211, 212).

### 5.2 Platelet-Treg cell interactions in ALI resolution

Platelets are instrumental in the resolution of inflammation (30). As the inflammatory response shifts toward healing, platelets release anti-inflammatory mediators like TGF-β, IL-10, and PGE2 (213). These factors help promote the polarization of macrophages to an M2 phenotype, which is associated with tissue repair and the resolution of inflammation (214). Moreover, platelets engage with regulatory T cells (Treg cells), fostering their differentiation and enhancing their anti-inflammatory effects (30). This interaction is thought to be critical for transitioning the immune response from a pro-inflammatory state to a reparative phase, thus aiding in the resolution of ALI. Additionally, platelet-Treg cell aggregation supports the shift of macrophages to an anti-inflammatory state, optimizing alveolar macrophage phagocytosis (215).

**Figure 3:**
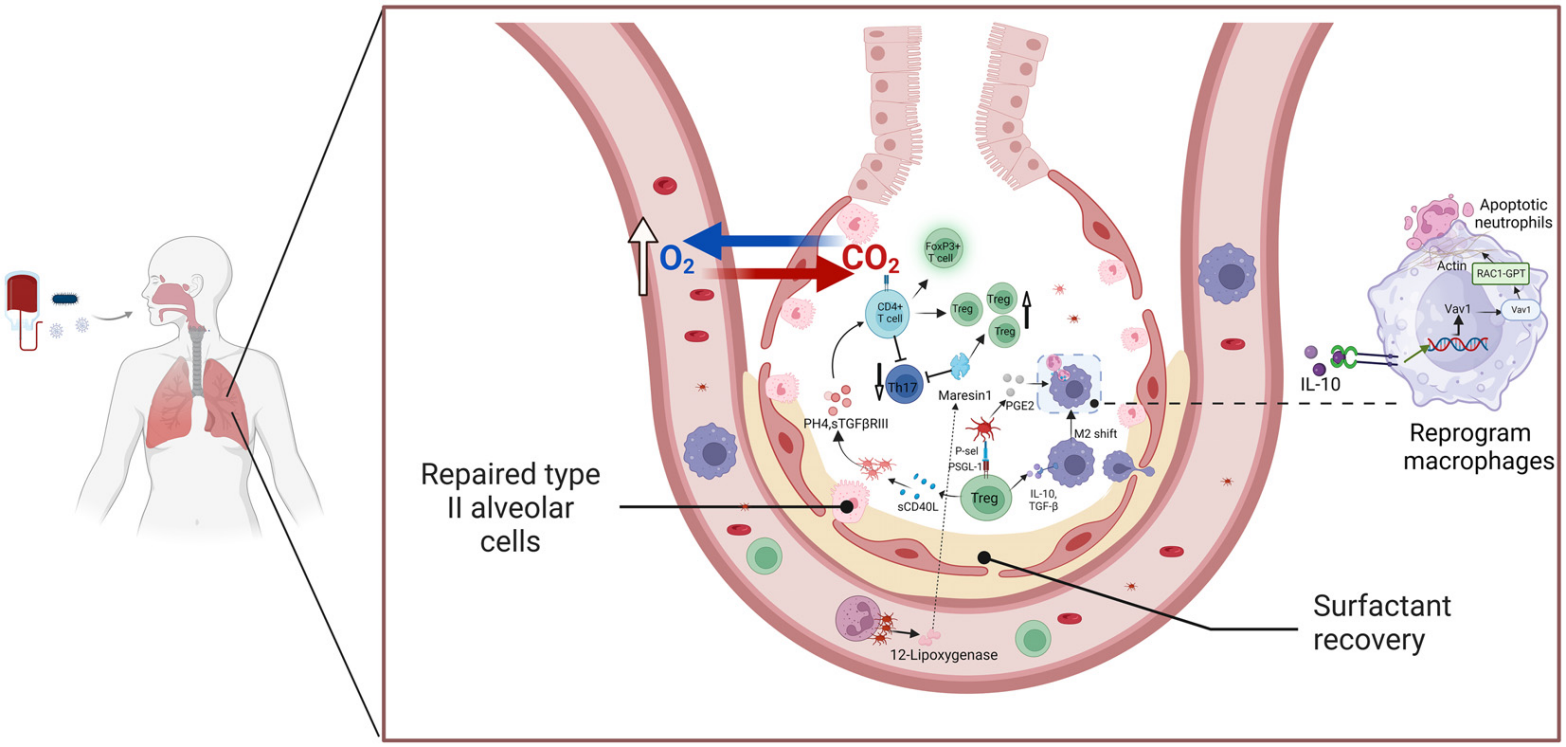
The platelets and Treg cells crosstalk mediate the resolution of ALI/ARD inflammation. This schematic illustration highlights the interaction between platelets and Tregs in facilitating the resolution of inflammation in ALI and ARDS. Activated platelets release key mediators such as TGF-β and PF4, which enhance Treg activation and function. In response, Tregs secrete anti-inflammatory cytokines, including IL-10 and TGF-β, which contribute to immunosuppression and the resolution of inflammation. These cytokines also promote macrophage polarization toward the M2 phenotype and assist in neutrophil apoptosis, aiding in the restoration of immune balance and tissue repair. The platelet-Treg crosstalk plays a crucial role in limiting excessive inflammation and promoting recovery in ARDS, highlighting its potential as a therapeutic target. Abbreviations: ALI, acute lung injury; ARDS, acute respiratory distress syndrome; TGF-β, transforming growth factor-beta; PF4, platelet factor 4; IL-10, interleukin-10; Treg, regulatory T cell.

Recent studies highlight that platelets play a role in the resolution of lung inflammation through their interactions with Tregs and macrophages (216) (**Figure 3**). Specifically, platelet P-selectin binds to PSGL-1 on T cells, forming platelet-Treg aggregates that promote CD4+ T cell differentiation into Tregs. Additionally, platelets are the primary source of soluble CD40L (sCD40L), which is released upon activation and further enhances platelet aggregation and activation. This interaction increases the number of Tregs in the lungs and facilitates the secretion of anti-inflammatory agents, contributing to inflammation resolution (215, 217). *In vitro* studies of platelet-T cell cocultures showed a marked increase in the proliferation and differentiation of the FoxP3+ Treg subset by day 3 (218). Platelet-derived PH4 promotes IL-10 secretion, a potent anti-inflammatory cytokine (218, 219). Platelets regulate T cell effector responses in a context-dependent manner through PF4-TGFβ interactions, with platelet coculture enhancing sTGFβRIII release, amplifying TGFβ signaling, and promoting CD4+ T cell effector functions (54). During the resolution phase of inflammation, Tregs in the lungs enhance macrophage internalization of apoptotic neutrophils through the Vav1-Rac1 pathway (211, 220). This process accelerates macrophage phagocytosis of apoptotic neutrophils and aids in the transition of macrophages from a pro-inflammatory to a reparative phenotype.

### 5.3 Platelet-derived mediators and their role in ALI recovery

Platelets can act directly or through mediators to regulate macrophage responses, enhancing bacterial clearance and reducing inflammation. Activated platelets release PGE2, prompting mononuclear macrophages to reduce TNF-α secretion while promoting the release of anti-inflammatory mediators and facilitating inflammation resolution (211, 221). A study by Tang et al. on acute liver injury repair provides an intriguing new perspective. Typically, neutrophils use ROS production to increase the release of NETs, which exacerbates inflammation (222). However, this study hypothesized that ROS activates AMP-activated protein kinase (AMPK), which shifts macrophages toward the repair phenotype, promoting liver repair (223). It remains an exciting question whether this mechanism also plays a role in ALI recovery. The balance between Th17 and Tregs is intricately linked to the resolution of pneumonia in ALI (224). In ALI/ARDS, neutrophil-platelet aggregates enhance the synthesis of MAResin1, a pro-resolving mediator released by platelets. MAResin1 regulates the balance between Th17 and Tregs, improving lung function during acute inflammation (225–227). However, the precise mechanism by which MAResin1 affects Th17/Treg differentiation remains unclear, and further studies are needed to unravel the complex mechanisms involved in ALI resolution.

In the resolution phase of ALI/ARDS, alveolar edema is significantly reduced, and lung function improves dramatically (228). During this period, platelets coordinate the resolution of pulmonary inflammation through interactions with Tregs and macrophages (216). P-selectin binds to PSGL-1 on Tregs, forming stable platelet-Treg aggregates, which release inflammatory mediators like TGF-β and IL-10 (229). sCD40L produced by Tregs mediates platelet activation, leading to the release of factors such as PH4 and sTGFβRIII, which enhance the number of Tregs in the lung (229). Moreover, platelet-Treg aggregates promote macrophage internalization of apoptotic neutrophils through the Vav1-Rac1 pathway, enhancing the phagocytic capacity of macrophages (211, 220). The balance between Th17 and Treg differentiation plays a crucial role in alleviating lung inflammation (224). In ALI/ARDS, neutrophil-platelet aggregates promote MAResin1 synthesis, which regulates Th17/Treg signaling and improves lung function during acute inflammation (225, 226, 230). Future studies should further elucidate the mechanisms by which platelets mediate MAResin1 signaling to regulate the Th17/Treg balance in ALI resolution.

## 6 Antiplatelet agents in the prevention and treatment of ALI/ARDS

### 6.1 Clinical relevance of antiplatelet therapy in ALI/ARDS

Acute critical illnesses, such as ALI/ARDS, pose significant clinical challenges and are associated with high morbidity and mortality rates. According to the Berlin definition, a study by Hendrickson reported mortality rates of 34.9%, 40.3%, and 46.1% for mild, moderate, and severe ARDS cases, respectively (231). Notably, the 28-day mortality rates for mild, moderate, and severe cases were 29.6%, 35.2%, and 40.9%, respectively (231). The pathological features of ALI/ARDS include increased pulmonary microvascular permeability, alveolar exudates rich in proteins, and pulmonary edema (232). Given the limited treatment options, such as mechanical ventilation and restrictive fluid management, there is an urgent need for safer and more effective pharmacological interventions (233).

### 6.2 Efficacy of antiplatelet agents in ALI management

Animal model studies have suggested that antiplatelet agents, including aspirin (ASA) and lipid compounds, could help alleviate ALI symptoms (234–236). Retrospective clinical observations have further supported the potential of antiplatelet agents in improving ARDS symptom management (234, 237, 238). Clinical data from Philip Toner et al. have also demonstrated the potential of ASA in the treatment of ALI/ARDS (239). However, a study assessing early ASA administration for ARDS found no significant difference in morbidity between the ASA and placebo groups by day 7 (240, 241). This suggests that ASA alone may not significantly alter the progression of ALI/ARDS. It has been hypothesized that ASA does not effectively reduce platelet-driven neutrophil recruitment or prevent neutrophil rolling on endothelial cells through P-selectin, which could contribute to suboptimal therapeutic outcomes (242, 243). Additionally, the efficacy of ASA in ALI/ARDS treatment may be influenced by factors such as dosage and timing of administration (244, 245). The relatively small number of positive control patients in the ARDS trial at day 7 may have limited the study’s power to detect the full potential of ASA (240, 246). Thus, the results of the LIPS-A study could have been improved with a larger sample size.

It is important to emphasize that platelets play a crucial role in maintaining the integrity of the alveolar-capillary barrier and in promoting alveolar recovery (240, 246). Recent research has revealed that, in addition to their well-established production in the bone marrow, platelets can also originate from megakaryocytes (MKs) residing in the lungs (247). These lung-derived MKs serve as an extramedullary site of thrombopoiesis, contributing to platelet homeostasis under both physiological and pathological conditions (248). Unlike bone marrow-derived platelets, those produced within the pulmonary microenvironment may possess specialized functional properties, particularly in immune regulation (249). Studies suggest that lung MKs not only replenish platelet levels but also actively influence endothelial integrity, neutrophil trafficking, and macrophage polarization (250). These findings indicate that lung-derived platelets may play a unique role in ARDS pathogenesis, with potential contributions to both inflammatory responses and tissue repair processes. As lung-resident platelets exhibit distinct immune-regulatory roles, systemic antiplatelet therapies may differentially impact pulmonary and circulating platelet populations (251). In particular, lung-derived platelets may interact directly with the alveolar microenvironment, influencing local immune responses and endothelial stability, which could have critical implications for ARDS progression (31, 252). Understanding how these locally generated platelets contribute to immune homeostasis and tissue repair could lead to new therapeutic strategies that specifically target lung MKs and their platelet output. A more refined approach that considers the local effects of lung megakaryocytes and their platelets could improve the efficacy of pharmacological interventions in ARDS.

This emerging understanding has significant implications for antiplatelet therapies in ARDS, as systemic platelet inhibition may affect lung-resident platelet populations differently from circulating platelets. Tailoring therapeutic strategies to account for both systemic and lung-specific platelet activity could be crucial for optimizing treatment efficacy. Further investigation is needed to clarify the precise role of pulmonary MKs in ARDS and explore whether targeting lung-specific platelet production could offer novel therapeutic avenues for managing platelet-driven inflammation in ALI. In the later stages of tissue repair, once inflammation begins to resolve, continued use of antiplatelet agents could suppress these beneficial responses. While ASA and other antiplatelet agents have shown promise in ALI/ARDS treatment, the multifaceted role of platelets must be carefully considered, particularly the potential effects of pharmacological interventions at different stages of the disease.

The role of platelet activation in ARDS pathogenesis varies across distinct ARDS phenotypes, emphasizing the need for a precision medicine approach (253). Recent studies have classified ARDS into hyperinflammatory and hypoinflammatory subtypes, with the hyperinflammatory phenotype exhibiting heightened platelet activation and an increased risk of thromboinflammatory complications (254, 255). This suggests that antiplatelet strategies may be particularly effective in hyperinflammatory ARDS patients, whereas their benefit in hypoinflammatory cases remains uncertain (256). Furthermore, biomarker-based classification has enabled better patient selection in clinical trials, improving the efficacy of pharmacologic interventions in ARDS subpopulations (257). Emerging research also suggests that platelet count may serve as a biological marker for ARDS severity, providing a valuable tool for stratifying patients in future clinical studies (20). Incorporating ARDS subphenotyping into clinical practice could help optimize treatment outcomes and guide individualized therapies.

Beyond aspirin, recent investigations have identified additional antiplatelet strategies for ARDS, aiming to curb platelet-driven inflammation while maintaining essential hemostatic functions. P2Y12 receptor inhibitors, such as ticagrelor, have shown promise in attenuating platelet-neutrophil interactions and reducing immune thrombosis, thereby mitigating vascular injury associated with ARDS (258). Likewise, GP IIb/IIIa inhibitors may help regulate platelet aggregation, decreasing microvascular occlusion and improving pulmonary perfusion in severe cases (259). Moreover, pro-resolving lipid mediators, including aspirin-triggered lipoxins and resolvins, are being actively studied for their dual role in modulating platelet activity and facilitating inflammation resolution (260). Unlike traditional antiplatelet agents, which broadly suppress platelet function, these bioactive lipids appear to fine-tune platelet responses, reducing excessive inflammation while preserving their beneficial contributions to tissue repair (261). This selective mechanism makes them particularly compelling as potential therapeutic options for ARDS. Given the complexity of ARDS pathophysiology, a more structured approach to antiplatelet therapy is needed. Recent evidence suggests that the effectiveness of antiplatelet drugs may vary depending on the phase of ARDS progression (262). In the early inflammatory phase, targeting platelet activation could mitigate endothelial dysfunction, immune thrombosis, and neutrophil-driven tissue damage. However, during the resolution phase, an overly aggressive inhibition of platelet function might hinder tissue repair.

Beyond antiplatelet therapy, additional immunomodulatory and regenerative treatments are being explored for ARDS. Cytokine inhibitors, including IL-6 and TNF-α blockers, have shown potential in dampening excessive inflammation and improving oxygenation in severe cases (21). Mesenchymal stem cell (MSC) therapy has also emerged as a promising approach due to its ability to modulate immune responses, promote alveolar epithelial repair, and reduce fibrosis (256). Additionally, biologics targeting immune checkpoints and metabolic pathways involved in platelet-leukocyte interactions may present new therapeutic opportunities (21). While these treatments remain under investigation, their integration into ARDS management could enhance current strategies by complementing antiplatelet interventions, offering a more comprehensive approach across different disease stages.

### 6.3 Future directions for antiplatelet strategies

Emerging research suggests that combination therapy strategies, integrating multiple classes of antiplatelet and anti-inflammatory agents, may enhance therapeutic efficacy. By combining classical antiplatelet agents (e.g., P2Y12 inhibitors) with pro-resolving lipid mediators, it may be possible to suppress excessive inflammation while preserving the platelet-mediated resolution of ALI (263, 264). Moreover, precision medicine approaches that tailor antiplatelet interventions based on ARDS phenotypes and biomarkers may improve patient outcomes by optimizing drug selection and timing. These strategies warrant further investigation to determine their clinical feasibility and long-term benefits.

As research on platelet involvement in ARDS advances, it has become evident that antiplatelet interventions may need to be tailored to different stages of the disease. In the early inflammatory phase, dampening platelet-driven immune activation may help prevent endothelial dysfunction and excessive thrombosis, whereas in the later phase, a more controlled approach that preserves platelet-mediated tissue repair may be preferable (31, 176). Integrating a precision medicine approach-wherein biomarkers and ARDS phenotypes guide therapeutic decisions-could help identify patient subgroups most likely to benefit from antiplatelet interventions. Future studies should also focus on elucidating the potential role of lung-resident platelets in modulating the immune microenvironment of the alveoli and whether distinct platelet subtypes exist with differential contributions to ARDS progression. Future should explore how combining different antiplatelet agents or adjusting their administration timing can optimize their efficacy while minimizing potential adverse effects. Also should be focused on refining these therapeutic strategies, identifying optimal treatment windows, and determining which patient populations stand to benefit most from targeted antiplatelet interventions. In addition, to explore the potential synergies between antiplatelet agents and novel ARDS treatments, including immunotherapy and regenerative medicine, to optimize clinical outcomes.

## 7 Future directions for ALI/ARDS research and treatment

### 7.1 Platelet involvement in ARDS and its implications

The pathological hallmark of ALI/ARDS is the exacerbation of inflammation and disruption of the alveolar vascular endothelial barrier. Recent studies have underscored the critical role of platelets and their immunomodulatory functions in lung diseases, which can be compared to their role in thrombosis. Given that pulmonary megakaryocytes are the source of platelets, a key question arises: do platelets derived from these megakaryocytes have a distinct role in ALI/ARDS? Identifying functional and pathway differences between pulmonary megakaryocyte-derived platelets and myeloid platelets may be crucial in identifying novel markers for intervention and prognosis in ALI/ARDS, potentially offering new therapeutic strategies for lung diseases. Emerging data indicate that lung-resident megakaryocytes may give rise to platelets with distinct phenotypic and functional traits adapted to the pulmonary immune landscape. Investigating these differences could uncover localized regulatory mechanisms and aid in identifying platelet-influenced subtypes of ALI/ARDS.

Platelet-neutrophil aggregation plays a critical role in the inflammatory process of ALI/ARDS. Platelets recruit neutrophils to inflamed areas of the lung via the P-selectin-PSGL-1 axis. This interaction amplifies neutrophil-derived ROS and enhances the body’s inflammatory defense. Platelet activation plays a role in modulating NET formation, a process that, while beneficial in pathogen defense, may also drive immunothrombosis and tissue damage. Investigating how platelets influence NET dynamics could provide novel therapeutic approaches for ALI/ARDS, as previously described.

### 7.2 Advancing precision medicine in ARDS

Given the heterogeneity of ARDS, future studies should focus on identifying patient subgroups most likely to benefit from platelet-targeted interventions. Advances in molecular profiling and biomarker discovery could help refine precision medicine strategies, allowing for more individualized and effective therapeutic approaches (257). Additionally, ARDS subphenotyping has been shown to enhance clinical trial design, improving patient selection and minimizing variability in treatment responses (256). GWAS studies have further highlighted platelet-related pathways in ARDS, suggesting their potential as novel therapeutic targets (20). Integrating ARDS phenotyping into future clinical trials will be crucial for advancing precision medicine and improving patient outcomes.

### 7.3 Emerging therapeutic approaches for ARDS

The clinical management of ALI and ARDS has primarily focused on supportive care, including mechanical ventilation, fluid management, and nutritional support (265). However, there is a clear need for more targeted therapeutic approaches. While corticosteroids have been considered for their anti-inflammatory effects, their clinical use remains debated due to concerns over adverse effects such as delayed wound healing and increased infection risk.

Recent interest has emerged in using antiplatelet agents such as aspirin (ASA) to modulate platelet activity and neutrophil recruitment in ARDS. Clinical trials suggest that ASA may help alleviate ARDS symptoms by disrupting platelet-leukocyte interactions, though further studies are needed to clarify its full clinical benefit. Additionally, biological therapies targeting inflammatory cytokines, such as IL-6 inhibitors and TNF-α blockers, have demonstrated potential in reducing ARDS severity. Stem cell therapies represent another promising avenue, as they facilitate tissue repair and immune regulation, potentially accelerating inflammation resolution in ARDS (261, 266, 267).

Platelet-targeted therapies, designed to modulate platelet function and enhance their tissue-repairing roles, also hold significant promise (31, 252). Given the dual role of platelets in both fostering and resolving inflammation, optimizing the timing of platelet modulation in ALI/ARDS remains crucial. Some studies suggest that in later stages, platelet activity may support tissue repair, whereas early inhibition of platelet aggregation could help prevent further injury. Thus, a deeper understanding of the temporal dynamics of platelet activity is crucial to prevent inadvertent disruption of reparative immune processes. Future research should focus on stage-specific platelet behavior and determine optimal windows for intervention to enhance clinical outcomes while limiting potential adverse effects.

## 8 Conclusion

ALI/ARDS remains a challenging clinical syndrome with significant mortality and a lack of effective pharmacological treatments. Recent research has drawn attention to the multifaceted role of platelets- not only as contributors to inflammatory damage but also as regulators of immune resolution. Their interactions with neutrophils, endothelial cells, macrophages, and Tregs reveal a complex network that governs the progression and potential recovery of lung injury.

Recognizing the phase-specific functions of platelets, and how these differ among patient subgroups, may inform the design of more nuanced therapeutic approaches. The integration of platelet-related markers into ARDS subphenotyping frameworks holds promise for advancing individualized treatment. Further studies aimed at linking platelet biology to clinical phenotypes will be critical in translating these insights into practical strategies for care.
